# Supplementary material for: Novel Driver Strength Index highlights important cancer genes in TCGA PanCanAtlas patients
Source: PeerJ. 2022 Aug 11;10:e13860. doi: 10.7717/peerj.13860 (PMC9375969; doi:10.7717/peerj.13860)
Supplement: Supplemental Information 2 [file peerj-10-13860-s002.zip › COHORTS/genes plots/detailed/2021_8_16_14_9_distribution_events_detailed_3.pdf]

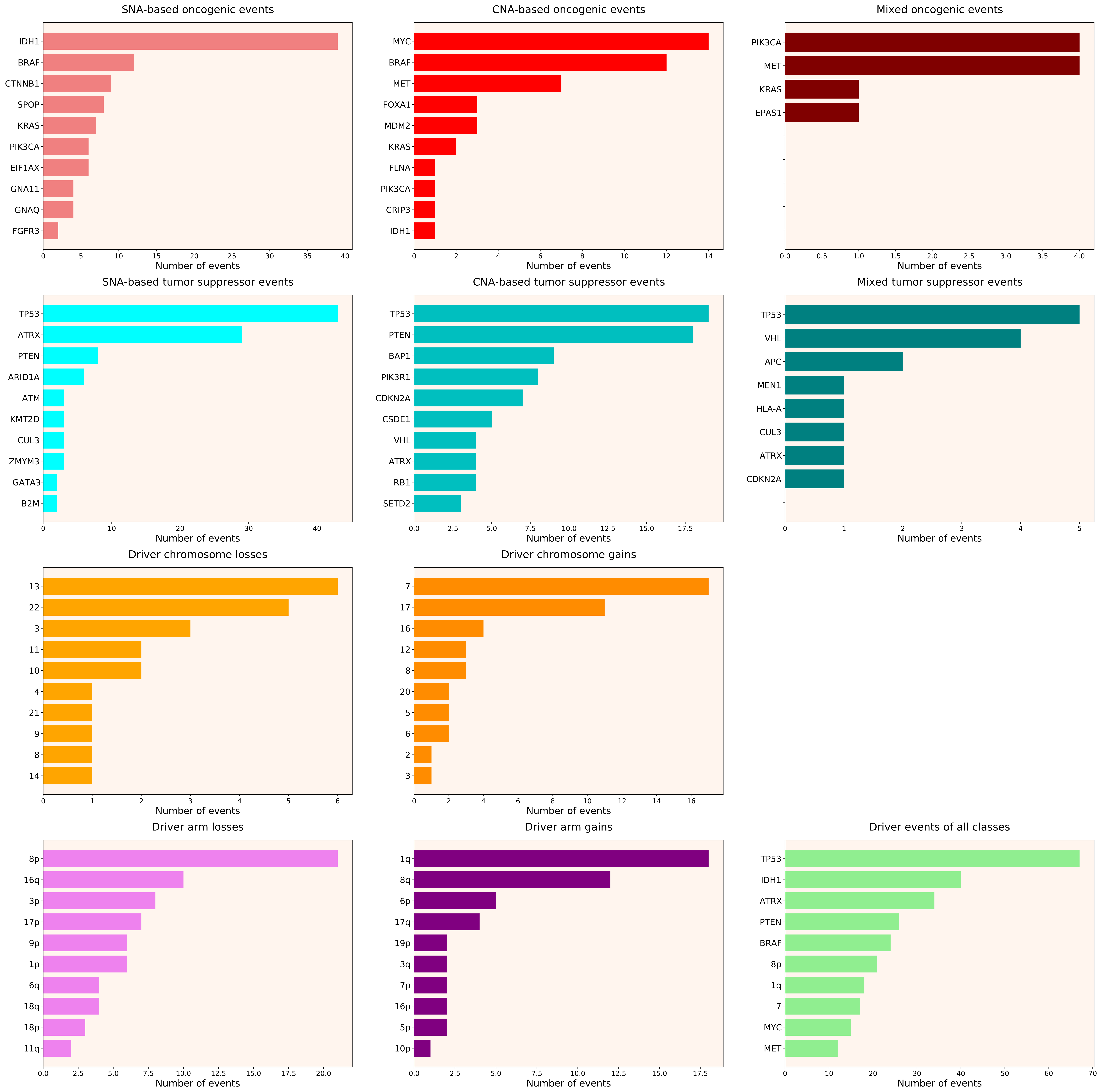

SNA-based oncogenic events
IDH1
BRAF
CTNNB1
SPOP
KRAS
PIK3CA
EIF1AX
GNA11
GNAQ
FGFR3
Number of events
CNA-based oncogenic events
MYC
BRAF
MET
FOXA1
MDM2
KRAS
FLNA
PIK3CA
CRIP3
IDH1
Number of events
Mixed oncogenic events
PIK3CA
MET
KRAS
EPAS1
Number of events
SNA-based tumor suppressor events
TP53
ATRX
PTEN
ARID1A
ATM
KMT2D
CUL3
ZMYM3
GATA3
B2M
Number of events
CNA-based tumor suppressor events
TP53
PTEN
BAP1
PIK3R1
CDKN2A
CSDE1
VHL
ATRX
RB1
SETD2
Number of events
Mixed tumor suppressor events
TP53
VHL
APC
MEN1
HLA-A
CUL3
ATRX
CDKN2A
Number of events
Driver chromosome losses
13
22
3
11
10
4
21
9
8
14
Number of events
Driver chromosome gains
7
17
16
12
8
20
5
6
2
3
Number of events
Driver arm losses
8p
16q
3p
17p
9p
1p
6q
18q
18p
11q
Number of events
Driver arm gains
1q
8q
6p
17q
19p
3q
7p
16p
5p
10p
Number of events
Driver events of all classes
TP53
IDH1
ATRX
PTEN
BRAF
8p
1q
7
MYC
MET
Number of events
